# Peripheral nervous system involvement accompanies central nervous system involvement in anti-N-methyl-D-aspartate receptor encephalitis

**DOI:** 10.3389/fimmu.2026.1865306

**Published:** 2026-08-12

**Authors:** Wenjie Wang, Lihui Zhu, Zhiting Li, Liran Qin, Hongying Liu, Li Wu, Youming Long, Xuelong Li, Xianliang Li, Jun Liu

**Affiliations:** 1 Department of Neurology, The Second Affiliated Hospital of Guangzhou Medical University, Institute of Neuroscience, Key Laboratory of Neurogenetics and Channelopathies of Guangdong Province and the Ministry of Education of China, Guangzhou, China; 2 Neurology Center, Guangzhou Fosun Chancheng Hospital of Guangdong Pharmaceutical University, Guangzhou, China

**Keywords:** anti-NMDAR encephalitis, autoimmune encephalitis, central nervous system, electroencephalogram, electromyography, electrophysiology, peripheral nervous system

## Abstract

**Background:**

Anti-N-methyl-D-aspartate receptor (NMDAR) encephalitis is the most common autoimmune encephalitis, primarily affecting the central nervous system (CNS) and typically presenting with neuropsychiatric symptoms. However, emerging evidence suggests that peripheral nervous system (PNS) involvement may also occur in some cases.

**Objective:**

To investigate the clinical and neuro-electrophysiological characteristics of PNS involvement in patients with positive anti-NMDAR antibodies in cerebrospinal fluid (CSF).

**Methods:**

We retrospectively reviewed all patients with CSF-confirmed anti-NMDAR encephalitis at the Second Affiliated Hospital of Guangzhou Medical University between 2016 and 2025. Collected data included initial symptoms and disease severity, CSF and serum test results, magnetic resonance imaging (MRI) and neuro-electrophysiological findings, primary treatment regimens, presence of coexisting autoimmune disorders, and clinical outcomes.

**Results:**

Among the 46 patients with anti-NMDAR encephalitis, 9 patients (19.6%) exhibited PNS involvement. The average age of onset in this subgroup with PNS involvement were significantly older than those with CNS involvement only (mean ± SD: 36.2 ± 18.9 years vs. 24.6 ± 12.4 years; *P* = 0.029). Limb weakness was significantly more frequent in the PNS involvement group (55.6% vs. 2.7%; *P* < 0.001). Electromyography (EMG) was performed in 18 patients (39.1%), of whom 10 patients (55.6%) showed abnormal findings. Among these, 1 patient exhibited active radiculopathy associated with motor neuron disease, 2 patients had isolated radiculopathy, and 1 patient presented with mixed polyneuropathy involving both motor and sensory functions. Cranial nerve involvement was observed in 2 patients (22.2%). In one case, PNS involvement was the initial symptom.

All patients received first-line immunotherapy. PNS involvement was not significantly associated with disease prognosis.

**Conclusion:**

PNS involvement in anti-NMDAR encephalitis is not uncommon and most frequently manifests as acute or subacute limb weakness. Neuro- electrophysiological abnormalities predominantly involve motor conduction pathways and proximal nerve roots. This is a single-center retrospective study with a small sample size. Large-sample prospective studies are needed to further validate the findings.

## 1 Introduction

Anti-N-methyl-D-aspartate receptor (NMDAR) encephalitis is an immune-mediated inflammatory disorder of the CNS that was first described in 2007 and has since become the most common type of autoimmune encephalitis (1, 2). The NMDAR, a glutamate receptor, serves as the principal excitatory synaptic protein in the CNS. Anti-NMDAR encephalitis typically manifests with acute or subacute onset (3–5). Approximately half of patients experience prodromal symptoms such as headache and fever within two weeks prior to disease onset. The most frequent clinical manifestations include behavioral and mental abnormalities (82.7%), followed by epileptic seizures (80.9%), cognitive impairment, and autonomic dysfunction (6, 7).

Most studies on anti-NMDAR encephalitis have centered on its CNS manifestations (8). Ligocki et al. reported that CSF can traverse the nerve root entry zones into the peripheral nerve fiber layer and reach distal axons (9). This anatomical pathway suggests that autoimmune-mediated inflammation may not be confined to the CNS but could also extend to peripheral nerves (9). Furthermore, studies have shown that functional ionotropic glutamate receptors, including NMDAR, are expressed on both axonal cylinders and myelin sheaths in the PNS, analogous to their presence in the CNS (10, 11). Excessive activation of these peripheral receptors leads to a marked influx of calcium ions into Schwann cell-associated axons and increased ribosomal activity. Notably, cleavage of NMDAR proteins within peripheral nerves itself may contribute to the encephalitic process. Thus, ionotropic glutamate receptors are likely to play a key role in both the pathophysiology of peripheral nerve injury and subsequent repair mechanisms in this disease context (12–14).

Although anti-NMDAR encephalitis predominantly presents with CNS symptoms, it can be accompanied by rare peripheral neuropathy, which has primarily been reported in some case reports (15–23). Given that these work were limited to case reports, peripheral neuropathy caused by comorbidities couldn’t be excluded. The PNS involvement exhibits heterogeneous electrophysiological patterns, including axonal damage (15, 17), demyelinating injury (23), or a mixed pattern (16, 18), with clinical manifestations ranging from flaccid paralysis to sensory abnormalities.

Current understanding of PNS involvement in anti-NMDAR encephalitis is largely based on isolated case reports. This study examines a systematically assembled cohort of patients with anti-NMDAR encephalitis complicated by peripheral neuropathy. We aim to investigate the more detailed and comprehensive clinical and electrophysiological characteristics of PNS involvement in anti-NMDAR encephalitis.

## 2 Methods

### 2.1 Study participants

This study included patients diagnosed with anti-NMDAR encephalitis at the Second Affiliated Hospital of Guangzhou Medical University University between January 2016 and January 2025. All cases fulfilled the diagnostic criteria of anti-NMDAR encephalitis outlined in the 2022 Chinese expert consensus on the diagnosis and management of autoimmune encephalitis (24) and the 2016 international expert consensus (25). Actually the diagnosis criteria are consistent with each other in these two consensuses. The indirect immunofluorescence method cell-based assay (CBA) was applied to test the CSF. When available, the serum samples, which were taken concomitantly to CSF samples, were tested using CBA.

### 2.2 Experimental design

A structured questionnaire was administered to collect relevant data. The collected information included demographic details, clinical manifestations, concomitant autoimmune diseases, biochemical indicators, electroneurography findings (including nerve conduction studies and needle-EMG and evoked potential), electroencephalogram (EEG) results, CSF analysis, cranial and spinal cord MRI characteristics, primary treatments administered, intensive care unit (ICU) admission, modified Rankin Scale (mRS) scores at diagnosis and final follow-up, as well as alternative differential diagnoses still under consideration by the clinicians at the last assessment.

Patients were followed up for a period of three to six months. The follow-up assessments included the persistence of initial symptoms, extent of recovery, and any disease-related functional impairment in the limbs. PNS involvement was defined as clinical symptoms consistent with peripheral neuropathy or radiculopathy, such as limb weakness or numbness, accompanied by diminished or absent tendon reflexes and negative pathological signs, or clinical evidence of cranial nerve injury (CN III-XII). Meanwhile, peripheral nerve damage may caused by diabetes mellitus, critical illness polyneuropathy (CIP), Guillain-Barré syndrome (GBS) and other rheumatic autoimmune diseases such as Sjögren’s syndrome, vasculitis have been excluded. CNS involvement was defined by the presence of central symptoms such as mental and behavioral abnormalities, limb or facial convulsions, involuntary movements, memory decline, altered consciousness, headache and dizziness. Acute or subacute onset was defined as the time from symptom onset to peak being less than three months, whereas progressive onset referred to a duration of more than three months between symptom onset and peak. Disease severity and outcome were assessed using the mRS. A significant treatment response was defined as a decrease of at least two points on the mRS or full recovery.

### 2.3 Statistical analysis

Continuous variables were expressed as means ± standard deviations if normally distributed, and as medians with interquartile ranges (IQR, Q1–Q3) if non-normally distributed. Categorical variables were presented as counts and percentages. Comparisons between groups were performed using the independent samples t-test for normally distributed continuous variables, the Wilcoxon rank-sum test for non-normally distributed continuous variables, and Fisher’s exact test for categorical variables. All tests were two-sided, and a P < 0.05 was considered statistically significant. No imputation was performed for missing data. All analyses were conducted using SPSS version 25.0.

### 2.4 Standard protocol approvals and patient consents

This study was approved by the Ethics Committee of the Second Affiliated Hospital of Guangzhou Medical University (Approval No. LYZX-2025-136-01) and was conducted in accordance with the principles of the 1964 Helsinki Declaration. As a retrospective case series, the requirement for informed consent was waived.

## 3 Results

### 3.1 Demographic features of patients with anti-NMDAR encephalitis

**Figure 1:**
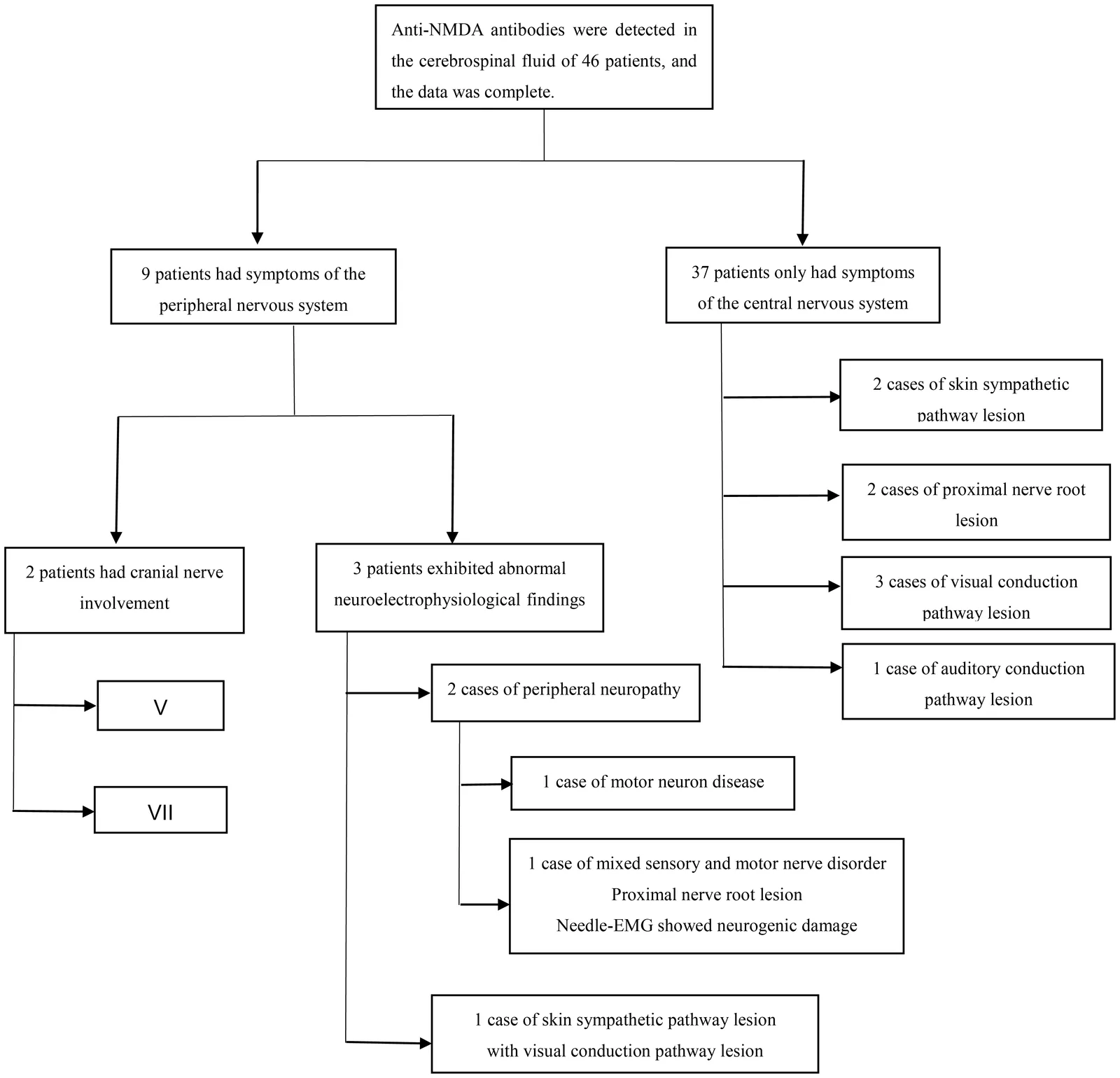
Diagram of anti-NMDAR encephalitis patients and PNS involvement. NMDAR, N-methyl-D-aspartate receptor; Needle-EMG, needle electromyography; PNS, peripheral nervous system.

A total of 46 patients were diagnosed with anti-NMDAR encephalitis. Among them, 9 patients presented with PNS symptoms, including flaccid paralysis, sensory abnormalities. Cranial nerve involvement—specifically of the trigeminal (fifth) and facial (seventh) nerves—was observed in 2 patients. Ten patients exhibited abnormal neuro-electrophysiological findings, such as impaired motor nerve conduction, mixed sensorimotor neuropathy, proximal nerve root lesions, or skin sympathetic response (SSR). (**Figure 1**).

The median age of disease onset in this study was 27 years (range, 4–47). Of the patients, 26 patients (56.5%) were male. Most cases (38/46; 82.6%) presented with acute or subacute onset. Nine patients (19.6%) exhibited PNS symptoms in combination with CNS symptoms, while 37 patients (80.4%) presented with CNS symptoms only. CSF analysis was performed in 44 patients at our institution; 2 additional patients underwent lumbar puncture at external facilities and were diagnosed with anti-NMDAR encephalitis. Abnormal CSF protein levels were observed in 17 patients (37.0%), with elevation in 10 patients (mean, 656.2 mg/L; IQR, 487–673 mg/L) and mild reduction in 7 patients. Elevated white blood cell counts were noted in 20 patients (43.5%). Hyperglycemia was present in 9 patients (19.6%), and hypochloremia in 12 patients (26.1%). Of the total cohort, 18 patients underwent EMG studies, which included nerve conduction studies, F-wave analysis, needle-EMG, and/or evoked potential testing. Among these 18 patients, 10 patients (21.7% of the total cohort) showed abnormal results. Specifically, abnormal nerve conduction was observed in 2 patients, abnormal F-wave results (manifested as decreased occurrence rate) were present in 3 patients, abnormal SSR results were noted in 4 patients, and abnormal evoked potentials were identified in 5 patients. Cranial MRI was performed in 41 patients, with parenchymal lesions detected in 20 patients. Among those with parenchymal involvement, 1 patient also showed meningeal involvement. Spinal MRI was performed in 7 patients, of whom 2 patients were found to have spinal meningitis. EEG was obtained in 45 patients, and abnormal findings were present in 43 patients (95.6%). Twelve patients (26.1%) required admission to the ICU due to disease severity. Three patients had concurrent immune disorders, all diagnosed with Hashimoto’s thyroiditis. Tumor markers were elevated in 12 patients (26.1%), and 1 patient had ovarian teratoma. At admission, mRS scores were greater than 3 in 27 patients (58.7%) and ranged from 0 to 2 in 19 patients. Regarding first-line immunotherapy, 45 patients (97.8%) received corticosteroids, 25 patients (54.3%) received intravenous immunoglobulin, 3 patients (6.5%) underwent plasmapheresis, and 6 patients (13.0%) received immunoadsorption. Nineteen patients received combined corticosteroid and intravenous immunoglobulin therapy. Second-line immunotherapy was administered to 8 patients (17.4%) with rituximab and 5 patients (10.9%) with cyclophosphamide; of these, 7 patients received concurrent corticosteroid therapy. At follow-up, clinical improvement was observed in 25 patients (54.3%), while the remainder experienced disease recurrence (**Table 1**).

**Table 1:** The clinical, laboratory test results, treatment and prognosis characteristics of all anti-NMDAR encephalitis cases.

Characteristics	Number of patients (%)	Median (IQR)
Age at onset, years		27 (16-36)
Male	26 (56.5%)	
Acute/subacute onset	38 (82.6%)	
CNS	46 (100%)	
PNS	9 (19.6%)	
CSF findings		
Elevated proteins Protein level (mg/L)	10 (21.7%)	656.2 (487-673)
Decreased proteins Protein level (mg/L)	7 (15.2%)	117.4 (100-142)
Pleocytosis White cell count (/mm3)	20 (43.5%)	27 (12-34)
Hyperglycorrhachia	9 (19.6%)	5.02 (5.57-5.68)
Hypochlorine	12 (26.1%)	117.5 (116.5-118.6)
Tumor indicators rise	12 (26.1%)	
Teratoma	1 (2.2%)	
Hashimoto’s thyroiditis	3 (6.5%)	
Abnormal EMG (total number=18)		
Motor nerve	2 (11.1%)	
Sensory nerve	1 (5.6%)	
F-wave	3 (16.7%)	
Needle-EMG	2 (11.1%)	
SSR	4 (22.2%)	
Evoked potentials	5 (27.8%)	
Cranial MRI(total number=41)		
Cranial parenchyma	20 (48.8%)	
Cranial parenchyma and meninges	1 (2.4%)	
Spinal cord MRI(total number=7)		
Myelitis	2 (28.6%)	
EEG(total number=45)		
Abnormality	43 (95.6%)	
Admission to ICU	12 (26.1%)	
mRS		
0-2	19 (41.3%)	
3-5	27 (58.7%)	
6	0 (0%)	
First-line immunotherapy		
Hormone	45 (97.8%)	
Immune globulin	25 (54.3%)	
Plasmapheresis	3 (6.5%)	
Immune adsorption	6 (13.0%)	
Rituximab	8 (17.4%)	
Long-course treatment	46 (100%)	
Oral corticosteroids	46 (100%)	
Oral MMF	2 (4.3%)	
Oral Cyclophosphamide	5 (10.9%)	
Oral azathioprine	2 (4.3%)	
Effect		
Improve	25 (54.3%)	
Relapse	21 (45.7%)	

NMDAR, N-methyl-D-aspartate receptor; IQR, interquartile range; CNS, central nervous system; PNS, peripheral nervous system; CSF, cerebrospinal fluid; EMG, electromyography; SSR, skin sympathetic response; MRI, magnetic resonance imaging; EEG, electroencephalogram; ICU, intensive care unit; mRS, modified Rankin Scale; MMF, mycophenolate mofetil.

“total number=18” in “Abnormal EMG (total number=18)” means only 18 patients underwent EMG; “total number=41” in Cranial MRI (total number=41) means only 41 patients underwent cranial MRI; “total number=7” in “Spinal cord MRI (total number=7)” means only 7 patients underwent spinal cord MRI; “total number=45” in “EEG(total number=45)” means only 45 patients underwent EEG.

### 3.2 Comparison of patients with or without PNS involvement in anti-NMDAR encephalitis

Among the 9 patients with PNS involvement in anti-NMDAR encephalitis, the mean age was 36.2 ± 18.9 years, compared to 24.6 ± 12.4 years in patients with isolated CNS involvement (*P* = 0.029). Within this group, 5 patients (55.6%) were male, and 7 patients (77.8%) presented with acute or subacute onset. Abnormal CSF biochemical markers were observed in 8 patients (88.9%). One patient initially presented with PNS symptoms manifesting as left limb weakness, prior to CNS symptoms. The majority of patients exhibited limb weakness (5/9, 55.6%), while 1 patient reported sensory abnormalities in the form of facial numbness. Another patient presented with clinical manifestations of mild left peripheral facial palsy, with positive eyelash sign, flattened nasolabial fold, and drooping corner of the mouth. Cranial MRI revealed parenchymal lesions in 5 patients (55.6%), and EEG showed abnormalities in 8 patients (88.9%). Three patients (33.3%) required transfer to the ICU, and 1 patients had concurrent viral encephalitis. All patients received corticosteroid therapy. In addition, 4 patients received intravenous immunoglobulin, 2 patients underwent immunoadsorption, 1 patient received plasmapheresis, and 1 patient was treated with rituximab. Despite treatment, 6 patients (66.7%) experienced disease recurrence. Elevated tumor markers were noted in 2 patients (22.2%). At admission, 3 patients (33.3%) had mRS scores exceeding 3, and these scores remained above 3 after treatment. (**Table 2**).

**Table 2:** Clinical, paraclinical and treatment characteristics of patients with or without PNS involvement in anti-NMDAR encephalitis patients.

Characteristics	PNS+CNS (n=9)	CNS (n=37)	P value
Age (mean ± SD)	36.2 ± 18.9	24.6 ± 12.4	0.029
Gender			
Female	4 (44.4%)	16 (43.2%)	1.000
Male	5 (55.6%)	21 (56.8%)	
Onset			
Acute/Subacute	7 (77.8%)	31 (83.8%)	0.645
Progressive	2 (22.2%)	6 (16.2%)	
Abnormal CSF			
Proteins	3 (33.3%)	14 (37.8%)	1.000
Pleocytosis	3 (33.3%)	17 (45.9%)	0.711
Hyperglucose	1 (11.1%)	8 (21.6%)	0.644
Chlorin	2 (22.2%)	10 (27.0%)	1.000
Symptom			
Muscular weakness	5 (55.6%)	1 (2.7%)	0.000
Headache	2 (22.2%)	3 (8.1%)	0.248
Feeling abnormality (pain, numbness)	1 (11.1%)	0	0.196
Mental and behavioral abnormality	1 (11.1%)	27 (73.0%)	0.001
Convulsions, tremors	3 (33.3%)	17 (45.9%)	0.711
Fever	0	4 (10.8%)	0.571
Bladder symptoms	1 (11.1%)	5 (13.5%)	1.000
Cranial MRI			
Cranial parenchyma	5 (55.6%)	15 (40.5%)	0.472
Cranial parenchyma and Meninges	0	1 (2.7%)	1.000
EEG			
Abnormal	8 (88.9%)	35 (94.6%)	0.488
Transfer to ICU	3 (33.3%)	9 (24.3%)	0.678
Hashimoto’s thyroiditis	1 (11.1%)	2 (5.4%)	0.488
First-line immunotherapy			
Hormonotherapy	9 (100.0%)	36 (97.3%)	0.180
Immune globulin	4 (44.4%)	21 (56.8%)	0.711
Rituximab	1 (11.1%)	7 (18.9%)	1.000
Immune adsorption	2 (22.2%)	4 (10.8%)	0.581
Plasmapheresis	1 (11.1%)	2 (5.4%)	0.488
Result			
Improve	3 (33.3%)	22 (59.5%)	0.264
Relapse	6 (66.7%)	15 (40.5%)	0.264
Tumor markers			
Rise	2 (22.2%)	10 (27.0%)	0.735
mRS (baseline)			
3-6	3 (33.3%)	24 (64.9%)	0.133

NMDAR, N-methyl-D-aspartate receptor; PNS, peripheral nervous system; CNS, central nervous system; SD, standard deviation; CSF, cerebrospinal fluid; MRI, magnetic resonance imaging; EEG, electroencephalogram; ICU, intensive care unit; mRS, modified Rankin Scale.

Thirty-seven patients exhibited symptoms exclusively related to the CNS, including mental and behavioral abnormalities, limb or facial convulsions, involuntary movements, memory decline, altered consciousness, headache and dizziness.

Within this group, 21 patients (56.8%) were male, and 31 patients (83.8%) presented with acute or subacute onset. CSF analysis was abnormal in 31 patients (83.8%). The majority demonstrated mental manifestations, noted in 27 cases (73.0%). Cranial MRI revealed cranial parenchymal involvement in 15 patients (40.5%), the temporal lobe was the most commonly affected region, followed by the hippocampus and parieto-occipital lobe, one of whom also showed meningeal enhancement. EEG abnormalities were observed in nearly all patients who were teken EEG examination. Due to disease severity, 9 patients (24.3%) required ICU admission. Homonotherapy was administered to 36 patients, after treatment 22 patients (59.5%) showed clinical improvement. Elevated tumor markers were present in 10 patients (27.0%); one patient was diagnosed with a hepatic hemangioma. At admission, mRS scores exceeded 3 in 24 patients (64.9%).

A statistically significant difference was observed in age at onset between the two groups. Clinically, limb weakness was significantly more frequent in the PNS involvement group (55.6% vs. 2.7%; *P* < 0.001), whereas CNS predominant symptoms such as mental and behavioral abnormalities were more common in the isolated CNS group (11.1% vs. 73.2%; *P* = 0.01). No significant intergroup differences were found in sex, mode of onset, CSF profiles, EEG results, cranial MRI findings, disease severity, treatment received, rates of ICU admission, and prognosis. (**Table 2**).

### 3.3 Clinical manifestations of CNS or PNS involvement in patients with abnormal EMG

Among all the patients, 18 of them underwent EMG tests, and 10 of them had abnormal results (55.6%). Three patients presented with symptoms of PNS involvement, manifested as weakness in their limbs, along with CNS involvement, mainly manifested as memory loss and convulsions. The results of the CSF tests showed that the protein levels slightly decreased in 3 individuals and increased in one individual. Three patients had elevated white blood cell counts. One patient suffered damage to the seventh cranial nerve, manifested as mild left peripheral focial palsy. The MRI of 6 patients showed damage to the brain tissue, and 1 patient also had spinal meningitis. The mRS scores of 2 patients were greater than 3 points. Four patients improved after treatment. (**Table 3**).

**Table 3:** Clinical characteristics of patients with abnormal EMG results in anti-NMDAR encephalitis.

Patient	Sex	Age	CNS/PNS	Cranial	Weakness	Paresthesia	Protein	White cell	MRI	mRS	Prognosis
1	Female	16	CNS	None	No	No	<100	2	Parenchyma	2	Relapse
2	Female	23	CNS+PNS	None	Yes	No	142.4	26	Parenchyma+myelitis	2	Relapse
3	Female	36	CNS	None	No	No	100	1	Parenchyma	3	Unchanged
4	Male	11	CNS	None	No	No	165.3	21	Parenchyma	4	Improve
5	Male	15	CNS	None	No	No	418,2	61	bilateral ethmoid sinusitis	3	Improve
6	Male	70	CNS+PNS	None	Yes	No	352	3	Parenchyma	1	Improve
7	Male	36	CNS	None	No	No	311.47	0	Parenchyma	3	Relapse
8	Female	14	CNS+PNS	VII	Yes	No	219.64	1	Normal	5	Relapse
9	Male	13	CNS	None	No	No	197.68	22	Normal	2	Unchanged
10	Female	26	CNS	None	No	No	515.94	3	Normal	1	Improve

EEG, electroencephalogram; NMDAR, N-methyl-D-aspartate receptor; CNS, central nervous system; PNS, peripheral nervous system; MRI, magnetic resonance imaging; mRS, modified Rankin Scale.

Among the 28 patients who did not undergo EMG, 21 cases had a mRS score of 3 or higher. Eighteen patients underwent EMG and 6 cases had a mRS score of 3 or higher. There was a statistically significant difference between them: the mRS score of patients who underwent EMG was lower than that of patients who did not undergo EMG (*P* = 0.007). (**Table 4**).

**Table 4:** Chi-square test for mRS in anti-NMDAR encephalitis patients with or without EMG examination.

Group	mRS 3-6	mRS 0-2	Total	P value
No EMG	21	7	28	0.007
EMG	6	12	18	
Total	27	19	46	

NMDAR, N-methyl-D-aspartate receptor; EMG, electromyography; mRS, modified Rankin Scale.

## 4 Abnormal manifestations of EMG in anti-NMDAR encephalitis

Eighteen patients underwent EMG tests and the EMG results of 10 patients were abnormal. One patient presented with active radiculopathy related to motor nerve disease: the amplitude of motor nerve conduction decreased, indicating axonal lesions. Positive sharp waves were observed in the needle-EMG. Two patients presented as isolated radiculopathy, with a decrease in the incidence of F-wave. One patient had a mixed type of polyneuropathy related to motor and sensory functions: there was a coexistence of demyelination and axonal damage of the motor and sensory nerves, with a decrease in the occurrence rate of F-wave, and abnormal phenomena such as positive sharp waves appeared in needle-EMG. The reduction in the occurrence rate of F-wave, the decrease in the amplitude of motor nerve conduction waves, and the appearance of positive sharp waves in needle-EMG were relatively common abnormalities.

Four patients showed no definitive waveforms on SSR examinzation, suggesting possible dysfunction of the autonomic nervous system. Among them, 2 cases experienced bladder dysfunction, which manifested as frequent urination and urinary incontinence.

Regarding the abnormality of evoked potentials, one patient exhibited an abnormality in brainstem auditory evoked potentials (BAEP), which was manifested as a lesion in the brainstem central segment of the left auditory pathway. Two patients showed that no waveforms were elicited in the right tibial nerve cortical lead of the somatosensory evoked potential (SEP), suggesting the damage in the central somatosensory pathway. Four patients showed abnormalities in visual evoked potentials (VEP). Three of them presented with bilateral demyelinating lesions, and one showed bilateral axonal damage. (**Table 5**).

**Table 5:** The results of patients with abnormal EMG.

Patient	MNCS	SNCS	F-wave	Needle-EMG	SSR	BAEP	VEP	SEP
1	Normal	Normal	Normal	–	No waveform	Normal	–	Normal
2	CMAP↓	Normal	Normal	Positive sharp wave	–	Normal	Amplitude↓	Central lesion
3	Normal	Normal	Occurrence↓	–	–	–	–	Normal
4	Normal	Normal	Occurrence↓	–	Normal	–	Normal	Normal
5	Normal	Normal	Normal	–	Normal	–	Latency↑	Normal
6	CMAP↓	SNAP↓	Occurrence↓	positive sharp+	–	–	–	–
	MCV↓	SCV↓		long-duration MUPs				
7	Normal	Normal	Normal	Normal	Normal	Latency↑	Latency↑	Normal
8	Normal	Normal	Normal	–	No waveform	–	Latency↑	Normal
9	–	–	–	–	No waveform	–	–	Normal
10	Normal	Normal	Normal	–	No waveform	–	–	Central lesion

MNCS, motor nerve conduction study; SNCS, sensory nerve conduction study; Needle-EMG, needle electromyography; CMAP, compound muscle action potential; SNAP, Sensory nerve action potential; SSR, skin sympathetic response; BAEP, brainstem auditory evoked potential; VEP, visual evoked potential; SEP, somatosensory evoked potential; MCV, motor nerve conduction velocity; SCV, sensory nerve conduction velocity; MUPs, motor unit potentials. The symbol “↓” represents that the corresponding column values decrease, and the symbol “↑” represents that the corresponding column values increase or prolong.

## 5 Typical case

A 70-year-old male was admitted to the hospital with a one-month history of weakness in the left lower limb, slurred speech, and intermittent convulsions. The patient had a history of foot injury caused by a metal nail two years prior. On admission, he presented with convulsions, trismus, and trunk rigidity, raising suspicion of tetanus, and was subsequently transferred to the ICU. CSF analysis revealed slightly decreased chloride levels (115.5 mmol/L) and mildly elevated white blood cell count (3/HP). GlyR1 and anti-NMDAR antibodies were positive. MRI demonstrated lacunar infarcts, while EEG showed mild to moderate abnormalities. Electrophysiological studies indicated mixed motor and sensory nerve impairment in four limbs, with reduced F-wave incidence and neurogenic changes on EMG (Case 6, **Table 5**). Clinical examination revealed weakness in both lower limbs and diminished tendon reflexes.

Following initial management for tetanus in the ICU, the patient was transferred to the neurology department for further treatment. He received methylprednisolone and intravenous immunoglobulin, underwent five sessions of plasma exchange, and was administered immunosuppressive therapy during his hospitalization. The patient eventually improved and was discharged, although residual weakness in both lower limbs persisted. Six months later, he returned for a follow-up visit due to bilateral lower limb numbness, the EMG reexamination still indicated multiple motor and sensory impairment in four limbs, which were markedly alleviated compared with previous findings.

## 6 Discussion

Anti-NMDAR encephalitis is the most common antibody-related autoimmune diseases, accounting for approximately 81% of autoimmune encephalitis cases (26). In the pathogenesis of anti-NMDAR encephalitis, antibodies in CSF play a more critical role than those derived from serum. The origin of anti-NMDAR antibodies within the CNS involves two main pathways: serum antibodies may penetrate the brain via a disrupted blood-brain barrier (BBB) (27, 28), and clonally expanded plasma cells in the CNS are proposed as an additional source of CSF anti-NMDAR antibodies (29, 30). Notably, these B cells are reported to originate from peripheral lymphoid organs (31, 32). This dual origin of pathogenic antibodies, combined with immune dysregulation involving both the CNS and peripheral immune system, provides a mechanistic basis for the potential concurrent involvement of the central and peripheral nervous systems in anti-NMDAR encephalitis. Consequently, beyond the classic CNS manifestations, patients with anti-NMDAR encephalitis may also present clinical features of peripheral neuropathy, reflecting the widespread immune-mediated damage across the neuraxis. Similarly, other antibody-mediated autoimmune encephalitides, such as those associated with glial fibrillary acidic protein (GFAP) (33–36) and IgLON5 (37–39) and leucinerich glioma-inactivated protein 1 (LGI1) (40–42) antibodies, may also exhibit peripheral neuropathy, further supporting the concept of widespread immune-mediated injury extending beyond the CNS in these disorders.

PNS damage is confirmed in anti-GFAP disease, occurring in approximately 20%-30% patients, owing to shared antigen expression in central glia and peripheral Schwann cells triggering immune injury (35, 43). By contrast, peripheral lesions are merely occasional and unvalidated in LGI1 and IgLON5 antibody disorders. Their target antigens mainly localize within the CNS, and rare peripheral manifestations are likely induced by secondary inflammatory responses (44).

Currently, most reported cases of PNS involvement in anti-NMDAR encephalitis have been described as isolated instances (21). We established a cohort to investigate PNS involvement in this condition. In our study, the predominant abnormal findings on EMG were pure motor or mixed motor-sensory or radiculopathy, were consistent with the neuroeletrophysiology damage patterns reported in previous cases (15–18, 23), whereas pure sensory neuropathy appears to be rare or unreported. Diseases potentially inducing peripheral nerve injury shoud be ruled out. CIP refers to damage of motor axons resulting from acute multiple organ dysfunction or systemic inflammation, typically observed in critically ill patients requiring mechanical ventilation (45). Since our patients exhibited peripheral neurological symptoms prior to ICU admission, CIP was considered unlikely. The lack of albuminocytological dissociation and atypical relevant clinical symptoms render GBS less probable. All patients denied medication history related to drug-induced neuropathy, and no individual suffered from diabetes mellitus, which makes diabetic peripheral neuropathy an unlikely etiology.

In patients with anti-NMDAR encephalitis, 20%–40% may have an underlying tumor, approximately 90% of which are teratomas (19). Paraneoplastic syndromes previously reported in association with anti-NMDAR encephalitis typically present with sensory neuropathy (46, 47). One of our patients had a teratoma without clinical manifestations or EMG findings suggestive of PNS involvement, the main clinical symptoms included headache accomanied by limbs convulsions. Some patients report a history of viral infection before disease onset (48, 49). Three patients in our cohort were diagnosed with concurrent viral infection, among whom one tested positive for herpes simplex virus (HSV) in the CSF. This virus can cause nerve root injuries (50). As for the clinical menifestations, 1 patient presented with dizziness, 1 patient had paroxysmal mouth corner twitching, and the third one suffered from aggravated headache accompanied by abnormal mental and behavioral symptoms. EMG revealed abnormal SSR in 2 patients, while the remaining one did not undergo EMG examination. All three cases were classified into CNS group. We speculated that these infectious agents might have played a role in triggering anti-NMDAR mediated autoimmunity, in addition to their own pathogenicity. Comparable findings have been documented in previous researches (51).

Peripheral nerve lesions can be observed in anti-NMDAR encephalitis, which may be attributed to the widespread distribution of NMDARs. These receptors are not only expressed in the neocortex, but also present in the anterior horn of the spinal cord (52). Some scholars have identified antibody-mediated motor neuron damage in autopsy specimens from patients with anti-NMDAR encephalitis (53). Meanwhile, activation of NMDARs may induce disorganized arrangement of myelin lamellae in the nervous system (12). The co-occurrence of NMDAR encephalitis and peripheral neuropathy deserves close clinical attention. This comorbid state may represent coexistence of distinct neuroimmune disorders or an overlapping neuroimmune syndrome.

Among anti-NMDAR encephalitis patients with abnormal evoked potentials, abnormalities in bilateral visual pathways were frequently observed, with demyelination being the most common pattern. Notably, these patients did not present with corresponding clinical symptoms.There were some cases of anti-NMDAR encephalitis combined with optic neuritis have been reported previously (54). Most patients presented with unilateral ocular involvement, while a small number involved bilateral lesions. It is speculated that anti-NMDAR antibodies may play a role in inducing demyelination-like lesions in the optic nerve. Whether optic neuritis serves as a manifestation of anti-NMDAR encephalitis or arises from concomitant autoimmune comorbidities remains to be further investigated. Abnormal results were found in 4 out of 11 patients undergoing SSR tests, indicating involvement of autonomic nerve function. Clinically manifest autonomic dysfunction occurs in approximately 51% of patients with anti-NMDAR encephalitis (55). The autonomic nervous system consists of complex neurons and pathways covering central and peripheral nerves (56). Skin biopsy of one anti-NMDAR encephalitis patient with autonomic dysfunction presented reduced foot intraepidermal nerve fiber density, consistent with small fiber neuropathy (57).

Notably, patients who did not undergo EMG tended to present with more severe symptoms at baseline. This may be attributed to the fact that some treating teams were unaware of PNS involvement and, in cases of severe clinical presentation, did not consider performing EMG. Although the number of patients who underwent EMG was limited, the positivity rate was relatively high. This suggests that among those not tested, a proportion may also have had subclinical or undetected PNS involvement.

The most common clinical symptoms in anti-NMDAR encephalitis are mental abnormalities, followed by epileptic seizures (6, 7), which is consistent with our findings. The average age of onset in patients with PNS involvement was 36 years, older than that in patients with isolated CNS involvement. Advanced age at disease onset may be associated with risk factors contributing to PNS involvement, including common metabolic diseases such as diabetes mellitus, as well as rheumatic autoimmune diseases like Sjögren’s syndrome and vasculitis. In this study, we recorded chronic diseases and other autoimmune comorbidities of the enrolled patients. Hashimoto’s thyoiditis was identified in three cases. Diseases that may cause impairment as mentioned above were not observed. Nevertheless, unidentified and unrecorded risk factors that may exert impacts on peripheral nerves cannot be completely excluded.

Limb weakness occured more frequently in patients with PNS involvement compared with isolated CNS involvement. In contrast, mental and behavioral abnormalities were relatively less common, the predominant clinical symptoms include headache, tremer, limb convulsion or memory loss. The potential mechanism may lie in NMDAR is highly expressed in prefrontal/limbic regions but scarce in peripheral nerves (58). When concurrent occurs, system pathological responsed PNS, relatively alleviating CNS impairment. This is just a tentative assumption awaiting further research confirmation.

Studies have reported that GFAP-positive patients with PNS involvement may exhibit bladder dysfunction (43), possibly due to cauda equina injury. In our cohort, six patients showed bladder dysfunction such as urinary or fecal incontinence, only one of whom had PNS involvement. Comparative analysis revealed that patients with PNS involvement were not more likely to develop bladder dysfunction. Furthermore, statistical analysis showed no significant difference in antibody titers between patients with and without PNS involvement.

Previous research has indicated that at least one-fifth of patients with autoimmune encephalitis show a positive response to immunotherapy (59, 60), which aligns with our findings. Nearly all patients in our cohort received first-line immunotherapy, and over 80% exhibited a reduction in their modified mRS score at discharge. In this study, the involvement of the PNS exerted no significant impact on patients’ prognosis. Further verifications based on larger sample size and more prospective design are required.

This study also has several limitations due to its retrospective single-center design and relatively small sample size. To be specific,

1. The sample size of the PNS involvement subgroup was relatively small (n=9), which may reduce the statistical power and increase the risk of type II errors. Therefore, negative or non-significant findings should be interpreted with caution to avoid overinterpretation and overstatement of the current results.
2. Not all patients received standardized and complete neuroelectrophysiological examinations. This may lead to potential selection bias and incomplete evaluation of subclinical peripheral nerve impairment. Whether the presence of PNS involvement affects patient prognosis requires further investigation with larger cohorts.
3. Patients with common causes of peripheral neuropathy were excluded according to strict clinical criteria. However, subtle or undetectable underlying peripheral nerve lesions cannot be completely ruled out. Further large-sample, prospective, and standardized electrophysiological studies are warranted.

In summary, anti-NMDAR encephalitis with PNS involvement can arise from multifactorial mechanisms and is often associated with a poor prognosis, though the difference didn’t reach statistical significance. In middle-aged and elderly patients presenting with unexplained limb weakness or other peripheral neurological symptoms accompanied by CNS involvement, the possibility of anti-NMDAR encephalitis should be considered in the differential diagnosis. Peripheral nerve manifestations may serve as an clinical indicator of the disease, and early electrophysiological testing can aid in its detection. Greater clinical attention should be paid to the occurrence of PNS involvement in anti-NMDAR encephalitis.

## Data Availability

The original contributions presented in the study are included in the article/supplementary material. Further inquiries can be directed to the corresponding author.
